# FOXO1-Induced miR-502-3p Suppresses Colorectal Cancer Cell Growth through Targeting CDK6

**DOI:** 10.1155/2023/2541391

**Published:** 2023-01-29

**Authors:** Hongwei Fan, Shuqiao Zhao, Rong Ai, Xuemin Niu, Junxia Zhang, Lin Liu

**Affiliations:** ^1^Department of Gastroenterology, Shijiazhuang People's Hospital, Shijiazhuang 050011, Hebei, China; ^2^Department of Pathology, Shijiazhuang People's Hospital, Shijiazhuang 050011, Hebei, China

## Abstract

Colorectal cancer (CRC) is the most common tumor of the digestive system and the third most common tumor worldwide. To date, the prognosis of CRC patients remains poor. It is urgent to identify new therapeutic targets for CRC. As a tumor suppresser, microRNA (miRNA) miR-502-5p is downregulated in CRC tissues. Nevertheless, the role of miR-502-3p in CRC is largely unclear. Besides, the transcript factor forkhead box protein O1 (FOXO1) could suppress the CRC cell growth. However, the effect of FOXO1 on miR-502-3p in CRC remains unknown. By contrast, cyclin-dependent kinases 6 (CDK6) promotes the CRC cell growth. Yet the regulatory effect of miR-502-3p on CDK6 in CRC has not been reported. Thus, the primary aim of this study was to investigate whether FOXO1 enhanced miR-502-3p expression to suppress the CRC cell growth by targeting CDK6. Here, RNA level and protein level were detected by quantitative reverse transcription-PCR (qRT-PCR) and western blot (WB), respectively. Besides, the cell growth was detected by Cell Counting Kit 8 (CCK8) assay. Moreover, the regulatory effect of FOXO1 on miR-502-3p or miR-502-3p on CDK6 was determined using dual-luciferase reporter gene (DLR) assay. Results revealed that miR-502-3p and FOXO1 were downregulated in CRC cells. Besides, miR-502-3p suppressed the CRC cell growth. Moreover, FOXO1 could increase the miR-502-3p level through facilitating *MIR502* transcription in CRC cells. In addition, miR-502-3p could suppress the CRC cell growth by targeting CDK6. These findings indicated that FOXO1 induced miR-502-3p expression to suppress the CRC cell growth through targeting CDK6, which might provide new therapeutic targets for CRC.

## 1. Introduction

CRC is the most common tumor of the digestive system and the third most common tumor worldwide [1–3]. In China, 55.5 thousand new CRC cases are reported and 28.6 thousand CRC patients die annually [4–6]. More noteworthy is that the incidence rate of CRC is still growing rapidly [4, 7]. What is worse is that the prognosis of CRC patients remains poor due to postoperative recurrence and metastasis, and the 5-year survival of stage IV patients with CRC is only 10% [8, 9]. Thus, it is urgent to seek new therapeutic targets for CRC to improve the prognosis of patients with CRC.

Numerous studies have revealed that miRNAs play critical roles in CRC. For instance, miR-17-5p facilitates tumorigenesis and metastasis of CRC through suppressing B-cell linker [10]. By contrast, miR-31 reduces serine/threonine kinase 40 (STK40) expression to improve radiosensitivity of CRC cells [11]. A previous study has indicated that miR-502-3p is downregulated in CRC tissues [12]. Besides, several studies have shown that miR-502-3p exerts an anticarcinogenic effect on gallbladder cancer, gastric cancer, and invasive pituitary adenoma. For example, long noncoding RNA (lncRNA) highly expressed in GBC (HGBC) promotes gallbladder cancer progression via sponging miR-502-3p [13]. Moreover, circular RNA ribosomal protein L15 (circ-RPL15) facilitates gastric cancer progression through inhibiting miR-502-3p [14]. In addition, lncRNA LINC00473 stimulates pituitary adenoma cell proliferation served as a competing endogenous RNA (ceRNA) of miR-502-3p [15]. However, the effect of miR-502-3p on CRC is largely unknown.

As a known transcription factor, FOXO1 is downregulated in CRC cells and prohibits the CRC cell growth [16–18]. Besides, FOXO1 could regulate miRNA expression. For example, FOXO1 enhances *MIR148A* transcription to increase miR-148a expression in hepatocytes [19]. Moreover, unacetylated FOXO1 translocates to the nucleus and promotes *MIR449A* transcription to elevate the miR-449a level [20]. Nevertheless, the regulatory effect of FOXO1 on miR-502-3p in CRC remains unclear.

CDK6 is a recognized cell cycle kinase facilitating cancer cell proliferation to promote cancer progression [21]. Besides, CDK6 is upregulated in CRC cells [22]. Moreover, CDK6 promotes CRC progression. A recent study has revealed that miR-500a-3p suppresses CRC progression through inhibiting aerobic glycolysis by targeting CDK6 [22]. By contrast, lncRNA CASC21 enhances the CRC cell growth by inducing CDK6 expression [23]. Yet the role of miR-502-3p in CDK6 expression in CRC has not been reported.

Therefore, the primary aim of the current study was to investigate whether FOXO1 enhanced miR-502-3p expression to suppress the CRC cell growth by targeting CDK6.

## 2. Methods and Materials

### 2.1. Cell Culture

Normal colonic mucosa cell line FHC cells and CRC cell line HT29 cells were obtained from the Cell Bank at the Chinese Academy of Sciences (Shanghai, China). Then, FHC and HT29 cells were cultured with Dulbecco's modified eagle's medium (DMEM) and 10% fetal bovine serum (FBS) (Gibco BRL, Grand Island, NY, USA) in a humidified incubator supplemented with 95% O_2_ and 5% CO_2_ at 37°C.

### 2.2. Cell Transfection

In this study, miRNA mimic, inhibitor, and vectors were transfected into HT29 cells using Lipofectamine 2000 (Invitrogen, Carlsbad, Calif, USA). Then, HT29 cells were collected and used for subsequent experiments at 48 hours post-transfection.

### 2.3. QRT-PCR

First, total RNA from HT29 cells were isolated using Trizol (Invitrogen). For mRNA detection, 1 *μ*g RNA was reverse transcribed by PrimeScript RT reagent Kit (Takara, Dalian, Liaoning, China). For miRNA detection, TaqMan miRNA assays (Applied Biosystems, Forest City, CA, USA) was utilized to reverse transcript 1 *μ*g RNA. Then qRT-qPCR analysis was carried out by the ABI 7500 fast real-time PCR system (Applied Biosystems) using SYBR Premix Ex Taq II ((Tli RNaseH Plus)) (Takara). Subsequently, the amount of target RNA was normalized to that of internal control (18 s or U6) and then the data were given by 2^−△△Ct^ relative to that of the control group. The primers used for qRT-PCR were listed as follows: miR-502-3p: forward: 5′-ACACTCCAGCTGGGAATGCACCTGGGCAAGGA-3′, reverse: 5′-CTCAACTGGTGTCGTGGA-3′; U6 forward: 5′-CTCGCTTCGGCAGCACA-3′, reverse: 5′-AACGCTTCACGAATTTGCGT-3′; FOXO1 forward: 5′-GGCAGCCAGGCATCTCATAA-3′, reverse: 5′-TTGGGTCAGGCGGTTCATAC-3′; 18 s forward: 5′-CCTGGATACCGCAGCTAGGA-3′, reverse: 5′-GCGGCGCAATACGAATGCCCC-3′.

### 2.4. CCK8 Assay

First, 1 × 10^4^ HT29 cells were collected in a well of the 96-well plate. Then, 10 *μ*L CCK8 solution (#C0038, Beyotime Biotechnology, Shanghai, China) was added into each well at a 1/10 dilution to incubate HT29 cells for 2 hours at 37°C. Next, Multiscan MK3 (Thermo Fisher Scientific, Waltham, MA, USA) was used to read the absorbance at 450 nm. Finally, the rate of HT29 cell proliferation was calculated based on the mean of optical density (OD) at 450 nm.

### 2.5. WB

First, total proteins were extracted from HT29 cells by RIPA lysis buffer (#P0013D, Beyotime Biotechnology). Then, 30 *μ*g protein was separated by SDS-polyacrylamide gel electrophoresis followed by the transfer onto a PVDF membrane (Millipore, Bedford, MA, USA). Next, 5% nonfat milk was used to block the membrane at room temperature (RT) for 1 hour and subsequently incubated with primary antibodies at 4°C overnight. Subsequently, Tris-buffered saline (TBS) supplemented with 0.1% Tween20 was utilized to wash the membrane three times followed by the incubation with second antibody at RT for 1.5 hour. Finally, the signals of target proteins were determined by the enhanced chemiluminescent (ECL) detection. The primary antibodies used for WB included FOXO1 antibody (1 : 1000, #ab52857, Abcam, Cambridge, UK), CDK6 antibody (1 : 1000, #ab179450, Abcam), and GAPDH antibody (1 : 15000, #KC-5G5, Aksomicks, Shanghai, China).

### 2.6. Bioinformatics Analysis

HumanTFDB database (https://bioinfo.life.hust.edu.cn/HumanTFDB#!/) was used to analysis potential FOXO1 binding sites on the promoter of *MIR502*. Besides, to mine targets of miR-502-3p, crosslinking-immunoprecipitation and high-throughput sequencing data of ENCORI database (https://starbase.sysu.edu.cn/index.php) were utilized.

### 2.7. Expression Vector Construction

To construct FOXO1 expression vector, the open reading frame (ORF) of *FOXO1* was cloned into the pcDNA 3.1 vector obtained from TaKaRa.

### 2.8. DLR Assay

The promoter of *MIR502*, wildtype (WT) CDK6 mRNA 3′UTR or mutant (MUT) CDK6 mRNA 3′UTR containing mutated miR-502-3p binding site was cloned into the luciferase reporter gene vector pGL3-basic. After cotransfection with pGL3-basic vectors and FOXO1 expression vector, with mimic NC or miR-502-3p mimic, respectively, luciferase activity of HT29 cells was detected by the Dual Luciferase Reporter Assay System (Promega, Madison, WI, USA).

### 2.9. Statistical Analysis

Data in the present study were present as mean ± standard deviation (SD). Besides, statistical differences were analyzed utilizing SPSS 20 software (SPSS Inc., Chicago, IL, USA). Briefly, the comparation between two groups was identified by the unpaired Student's *t*-test, while one way ANOVA was used for statistics among multiple groups. *P* < 0.05 was considered statistically significant.

## 3. Results

### 3.1. MiR-502-3p Is Downregulated in CRC Cell Line

**Figure 1:**
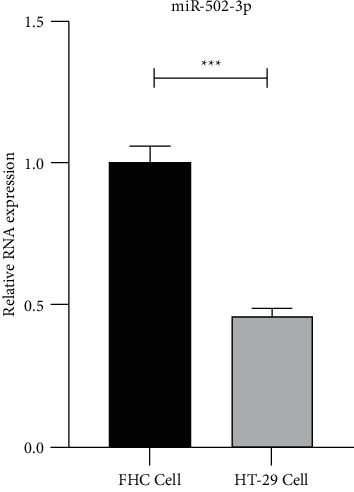
MiR-502-3p is downregulated in CRC cell line. The level of miR-502-3p in FHC cells and HT29 cells detected by qRT-PCR. *N* = 3. ^*∗∗∗*^*P* < 0.001.

First, the miR-502-3p level in HT29 cells was identified. Results of qRT-PCR showed that the level of miR-502-3p was reduced in HT29 cells compared to that in FHC cells (Figure 1), suggesting that miR-502-3p was downregulated in the CRC cell line. These results were consistent with those found in CRC tissues.

### 3.2. MiR-502-3p Suppresses CRC Cell Growth

**Figure 2:**
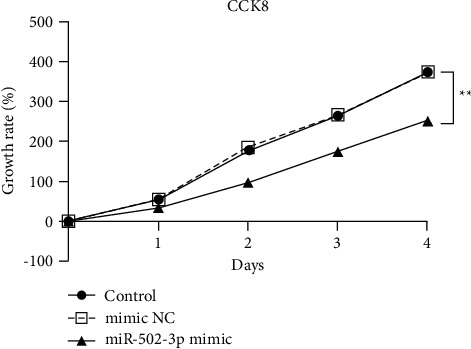
MiR-502-3p suppresses CRC cell growth. The growth rate of HT29 cells treated with or without miR-502-3p mimic detected by CCK8 assay. *N* = 3. NC: negative control. ^*∗∗*^*P* < 0.01.

Next, the effect of miR-502-3p on CRC cells was determined, and miR-502-3p mimic was used to overexpress miR-502-3p in HT29 cells. Results of CCK8 assay revealed that miR-502-3p overexpression dramatically decreased the HT29 cell growth rate compared to that of control HT29 cells (Figure 2). Besides, mimic NC had no effect on the HT29 cell growth rate (Figure 2). Abovementioned data suggested that miR-502-3p suppressed the CRC cell growth.

### 3.3. FOXO1 Elevates miR-502-3p Level through Facilitating *MIR502* Transcription in CRC Cells

**Figure 3:**
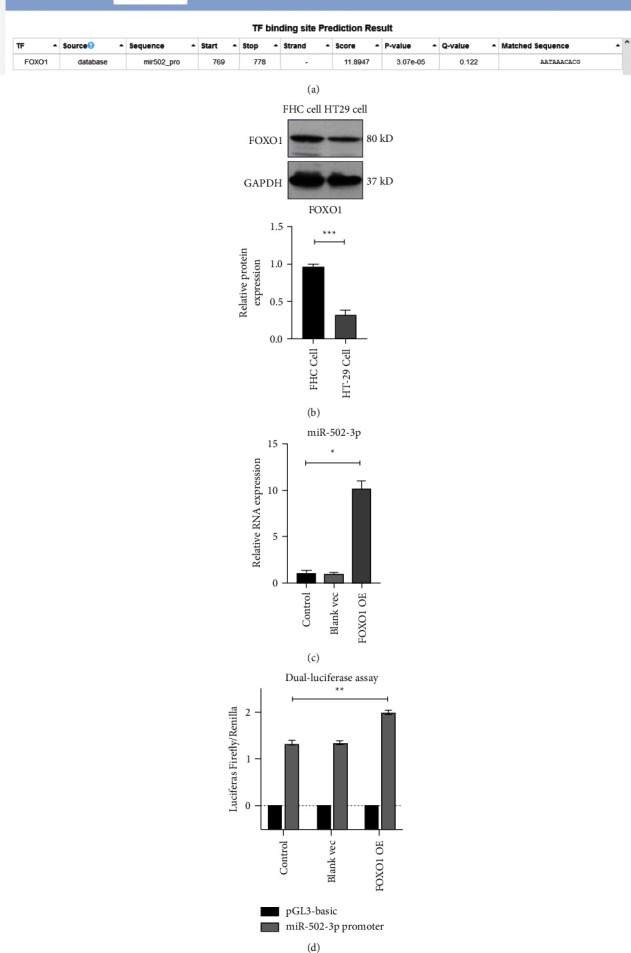
FOXO1 elevates miR-502-3p level through facilitating *MIR502* transcription in CRC cells. (a) Potential FOXO1 binding site on *MIR502* promoter analyzed using HumanTFDB database. (b) The protein level of FOXO1 in FHC cells and HT29 cells detected by WB. (c) The level of miR-502-3p in HT29 cells transfected with or without FOXO1 expression vector identified by qRT-PCR. (d) Luciferase activity of HT29 cells co-transfected with reporter gene vectors containing the promoter of *MIR502* and FOXO1 expression vector. Vec: expression vector; OE: overexpression. *N* = 3. ^*∗*^*P* < 0.05, ^*∗∗*^*P* < 0.01, ^*∗∗∗*^*P* < 0.001.

The pri-miR-502 is a transcript from *MIR502.* Moreover, potential FOXO1 binding site was found on the promoter of *MIR502* by bioinformatics analysis (Figure 3(a)). Consistent with the miR-502-3p level, the protein level of FOXO1 was also reduced in HT29 cells compared to that in FHC cells (Figure 3(b)). These results suggested that FOXO1 might regulate the miR-502-3p level in HT29 cells.

Next, results of qRT-PCR confirmed that FOXO1 overexpression by transfection of FOXO1 expression vector upregulated the miR-502-3p level in HT29 cells (Figure 3(c)). Besides, results of DLR assay indicated that the luciferase activity of HT29 cells transfected with pGL3-basic vectors containing the promoter of *MIR502* was increased by transfection of FOXO1 expression vector, while the blank expression vector had no effect on the luciferase activity of HT29 cells (Figure 3(d)). Thus, these data suggested that FOXO1 could elevate the miR-502-3p level through facilitating *MIR502* transcription in CRC cells.

### 3.4. MiR-502-3p Targets CDK6 in CRC Cells

**Figure 4:**
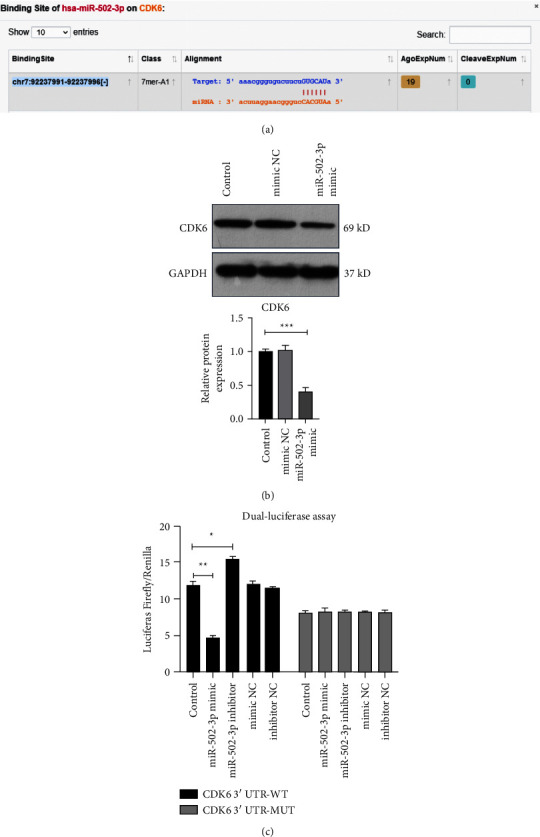
MiR-502-3p targets CDK6 in CRC cells. (a) miR-502-3p binding site on CDK6 mRNA 3′ UTR analyzed using ENCORI database. (b) The protein level of CDK6 in HT29 cells treated with or without miR-502-3p mimic detected by WB. (c) Luciferase activity of HT29 cells co-transfected with reporter gene vectors containing the binding site of miR-502-3p on CDK6 mRNA 3′ UTR and miR-502-3p mimic or inhibitor. *N* = 3. NC: negative control; WT: wildtype; MUT: mutant. ^*∗*^*P* < 0.05, ^*∗∗*^*P* < 0.01, ^*∗∗∗*^*P* < 0.001.

Bioinformatics analysis showed that CDK6 should be the target of miR-502-3p (Figure 4(a)). Next, results of WB confirmed that miR-502-3p overexpression dramatically reduced the CDK6 protein level in HT29 cells (Figure 4(b)). Besides, mimic NC had no effect on the CDK6 protein level in HT29 cells (Figure 4(b)).

To further identify the regulatory effect of miR-502-3p on CDK6, DLR assay was performed. Results showed that the luciferase activity of HT29 cells transfected with pGL3-basic vector containing the WT CDK6 mRNA 3′ UTR was reduced by miR-502-3p mimic whereas elevated by miR-502-3p inhibitor (Figure 4(c)). However, the luciferase activity of HT29 cells transfected with pGL3-basic vector containing the MUT CDK6 mRNA 3′ UTR with mutated miR-502-3p binding site was not regulated by miR-502-3p mimic or inhibitor (Figure 4(c)). Moreover, the luciferase activity of HT29 cells transfected with pGL3-basic vector was not affected by mimic NC and inhibitor NC (Figure 4(c)). As CDK6 could promote the CRC cell growth [23], these results together suggested that miR-502-3p should suppress the CRC cell growth through targeting CDK6.

## 4. Discussion

This study revealed the mechanism of miR-502-3p regulating the CRC cell growth. First, miR-502-3p was downregulated in CRC cells and suppressed the CRC cell growth. Second, FOXO1 could elevate the miR-502-3p level through facilitating *MIR502* transcription in CRC cells. Third, miR-502-3p should suppress the CRC cell growth by targeting CDK6.

Numerous studies have demonstrated the anticarcinogenic effect of miR-502-3p. For instance, lncRNA-HGBC facilitates gallbladder cancer progression through sponging miR-502-3p [13]. Besides, circ-RPL15 promotes gastric cancer progression by suppressing miR-502-3p [14]. Similarly, circDLST activates NRAS/MEK1/ERK1/2 pathway to aggravate gastric cancer progression via sponging miR-502-3p [24]. Moreover, lncRNA LINC00473 enhances pituitary adenoma cell proliferation serving as a ceRNA of miR-502-3p [15]. However, the role of miR-502-3p in CRC has not been explored. Therefore, this study for the first time revealed the anticarcinogenic effect of miR-502-3p in CRC, which is consistent with the role of miR-502-3p in other cancers.

The current study had indicated that the FOXO1 protein level was downregulated in CRC cells. Nevertheless, the mechanism regulating the FOXO1 protein level in CRC remains unclear. Several studies have revealed that FOXO1 protein expression is reduced by miRNAs in CRC. For example, miR-544 facilitates CRC progression through decreasing FOXO1 protein expression [25]. Besides, miR-135b decreases sensitiveness of oxaliplatin by reducing the FOXO1 protein level in CRC cells [17]. Moreover, miR-183-5p stimulates angiogenesis through suppressing FOXO1 protein expression in CRC [26]. In addition, miR-96 promotes CRC cell proliferation via downregulating the FOXO1 protein level [27]. Thus, the FOXO1 protein level might be reduced by miRNAs in CRC cells.

CDK6 could be regulated by miRNAs in various cancers. A previous study has revealed that miR-204 suppresses nonsmall cell lung cancer (NSCLC) progression through downregulating the CDK6 level [28]. Similarly, a recent study has indicated that miR-370-3p restrains the progression of ovarian cancer by reducing CDK6 expression [29]. Moreover, miR-576 represses CDK6 expression to promote bladder cancer cell proliferation [30]. In addition, miR-186 decreases the CDK6 level to inhibit the prostate cancer cell growth [31]. In CRC, both miR-539-5p and miR-500a-3p depress CRC progression via targeting CDK6 [22, 23]. Nevertheless, the effect of miR-502-3p on CDK6 is largely unknown. Therefore, this study for the first time revealed the inhibitory role of miR-502-3p in CDK6 during CRC progression.

However, there are still some limitations in the current study. For example, the association between FOXO1 and *MIR502* promoter should be further identified by chromatin immunoprecipitation (ChIP), while the interaction of miR-502-3p and CDK6 mRNA should be further determined using miRNA pulldown. In addition, the effect of miR-502-3p on the CRC cell growth should be confirmed by *in vivo* study performed in nude mice.

## 5. Conclusion

In summary, the current study revealed that downregulated miR-502-3p suppressed the CRC cell growth. Besides, FOXO1 could increase the miR-502-3p level through facilitating *MIR502* transcription in CRC cells. Moreover, miR-502-3p should suppress the CRC cell growth by targeting CDK6. These findings indicated that FOXO1 induced miR-502-3p expression to suppress the CRC cell growth through targeting CDK6, which might provide novel therapeutic targets for CRC.

## Data Availability

The data used to support the findings of this study are included within the article.
